# Effects of supplements differing in fatty acid profile to late gestational beef cows on cow performance, calf growth performance, and mRNA expression of genes associated with myogenesis and adipogenesis

**DOI:** 10.1186/s40104-021-00588-w

**Published:** 2021-06-14

**Authors:** Taoqi Shao, Frank A. Ireland, Joshua C. McCann, Daniel W. Shike

**Affiliations:** Department of Animal Sciences, University of Illinois at Urbana-Champaign, Urbana, IL USA

**Keywords:** Beef cattle, Fatty acids, Fetal programming, Late gestation, mRNA expression

## Abstract

**Background:**

Maternal nutrition during gestation affects fetal development, which has long-term programming effects on offspring postnatal growth performance. With a critical role in protein and lipid metabolism, essential fatty acids can influence the development of muscle and adipose tissue. The experiment investigated the effects of late gestation supplements (77 d prepartum), either rich in saturated and monounsaturated fatty acids (CON; 155 g/cow/d EnerGII) or polyunsaturated fatty acids (PUFA; 80 g/cow/d Strata and 80 g/cow/d Prequel), on cow performance and subsequent calf growth performance as well as mRNA expression in longissimus muscle (LM) and subcutaneous adipose tissue at birth and weaning.

**Results:**

There was no difference (*P* ≥ 0.34) in cow body weight (BW) or body condition score from pre-supplementation through weaning. Relative concentrations of C18:3n-3 and C20:4n-6 decreased (*P* ≤ 0.05) to a greater extent from mid-supplementation to calving for PUFA compared with CON cows. Cow plasma C20:0, C20:5n-3, and C22:6n-3 were increased (*P* ≤ 0.01) in PUFA during supplementation period. At birth, PUFA steers had greater (*P* = 0.01) plasma C20:5n-3. No differences (*P* ≥ 0.33) were detected in steer birth BW or dam milk production, however, CON steers tended (*P* = 0.06) to have greater pre-weaning average daily gain and had greater (*P* = 0.05) weaning BW compared with PUFA. For mRNA expression in steers: *MYH7* and *C/EBPβ* in LM increased (*P* ≤ 0.04) to a greater extent from birth to weaning for PUFA compared with CON; *MYF5* in LM and *C/EBPβ* in adipose tissue tended (*P* ≤ 0.08) to decrease more from birth to weaning for CON compared with PUFA; *SCD* in PUFA adipose tissue tended (*P* = 0.08) to decrease to a greater extent from birth to weaning than CON. In addition, maternal PUFA supplementation tended (*P* = 0.08) to decrease *MYOG* mRNA expression in LM and decreased (*P* = 0.02) *ZFP423* in adipose tissue during the pre-weaning stage.

**Conclusions:**

Late gestation PUFA supplementation decreased pre-weaning growth performance of the subsequent steer progeny compared with CON supplementation, which could have been a result of downregulated mRNA expression of myogenic genes during pre-weaning period.

**Supplementary Information:**

The online version contains supplementary material available at 10.1186/s40104-021-00588-w.

## Background

Fetal programming is the response to a specific challenge to the organism during a critical developmental period that leads to persistent effects [1]. In the last few years, fetal programming research has been focusing on specific nutrients, like fatty acids [2–5] and one-carbon metabolites [6–8]. By supplementing dietary fatty acids, the fatty acid profile of blood and tissues of the dams can be altered [9]. Meanwhile, maternal circulating fatty acids can be transferred to the fetus through placenta and alter the fatty acid profile of the fetus [10]. Therefore, supplementing different fatty acids to dams can modify the fatty acids transferred to the fetus. Fatty acids, especially essential fatty acids (EFAs), are important ligands for regulating protein and lipid metabolism, which can influence the development of fetal muscle and adipose tissue during critical periods. However, there is still lack of knowledge on whether the fetal fatty acids or modified dam metabolism would cause the fetal programming effects on the offspring.

Recent studies [2, 5] conducted under spring-calving and hay-based systems reported supplementation of Ca salts of polyunsaturated fatty acids during the last 90 days of gestation improved offspring growth performance during finishing phases and carcass characteristics. A series of sheep studies indicated that lamb performance was not only affected by lamb diet, but also influenced by maternal fatty acid supplementation during late gestation [4, 11]. It was also reported that late gestation supplementation of n-3 fatty acids to ewes increased mRNA expression of regulator of lipid mediator formation in fetal liver [12]. In cell studies, the function of Peroxisome proliferator-activated receptor gamma (PPARG), which is the key gene for adipogenic differentiation [13], is known to be stimulated by n-6 fatty acids such as C18:2 [14, 15]. However, n-3 fatty acids, especially C20:5n-3 (EPA) and C22:6n-3 (DHA), activate PPARα, which leads to increased transcription of lipolytic genes and decreased transcription of lipogenic genes [16]. It was also reported that dietary n-3 fatty acids intervention is critical for the development and function of bovine *Longissimus* muscle (LM) [17]. Human studies revealed that changes of maternal PUFA status during late gestation altered childhood body composition in young ages [18–20]. However, little is known about the mechanism of different responses in ruminants, especially beef cattle under fall-calving grazing systems. Therefore, the objective of the current study was to investigate the effects of supplementation differing in fatty acid profile to late gestation beef cows on cow performance, subsequent steer progeny growth performance during pre-weaning period, and mRNA expression in LM and subcutaneous adipose tissue. We hypothesized that cows would have similar performance since supplements were isocaloric and isonitrogenous and terminated at calving, while steers from dams supplemented with PUFA would have greater growth performance due to upregulated myogenesis and adipogenesis by fetal programming of EFAs.

## Materials and methods

Experimental animals were managed according to the guidelines recommended in the Guide for the Care and Use of Agricultural Animal in Agricultural Research and Teaching (Federation of Animal Science Societies, 2010). All experimental procedures were approved by the Institutional Animal Care and Use Committee of the University of Illinois (IACUC # 18109).

### Experiment design, animals, and diets

Ninety-six, fall-calving beef cows (body weight [BW] = 601 ± 76 kg) from Dixon Springs Agricultural Center, Simpson, IL were utilized for this experiment. Prior to supplementation (d − 77), cows were stratified by BW, body condition score (BCS), age, and fetal sex (64 male and 32 undetermined), and allotted into 8 predominately endophyte-infected tall fescue pastures with 12 cows/pasture (*n* = 4 pastures/treatment) and pasture groups were randomly assigned to 1 of 2 treatments. Each group of cows were rotated between two pastures every 2 weeks, with average stocking density of 3.0 cows per hectare. Forage availability was measured with rising plate meter at each rotation [21]. Each cow was supplemented 0.77 kg dry matter (DM)/d soybean hulls mixed with either 80 g DM/d Strata + 80 g DM/d Prequel (PUFA, rich in linoleic acid, eicosapentaenoic acid, and docosahexaenoic acid) or 155 g DM/d EnerGII (CON, rich in palmitic and oleic acids) for 77 ± 6 d prepartum (Table 1). Supplementation period began July 2. Calcium salts of fatty acids (Strata, Prequel, and EnerGII) were provided by Virtus Nutrition LLC, Corcoran, CA. The two diets were designed to be isocaloric and isonitrogenous. Nutritional and fatty acid profiles of ingredients fed to cows during late gestation are presented in Table 2. Cows were supplemented 3 times a week (Monday, Wednesday, and Friday) in 3 portable metal bunks (3.66 × 0.76 m, accessible from both sides) per group during supplementation period. Supplements were typically ingested by cows within 15 min. Cows were also weighed and assigned BCS at the middle of supplementation (d − 42), the end of the gestation (d − 18), within 1 week post-calving (5 ± 2.4 d post-calving), at artificial insemination (AI) pregnancy determination (d 113), and overall-pregnancy determination (d 186, same day as weaning) to monitor animal performance. Once weekly, cows that had calved and their calves were removed from the treatment groups, comingled to a common pasture, and managed as a single contemporary group from that point forward. Cows were supplemented with 2.27 kg/d/cow of dried distillers grains with solubles (DDGS; DM 81.2%, crude protein [CP] 30.4%, crude fat 10.6%, neutral detergent fiber [NDF] 32.9%, and acid detergent fiber [ADF] 11.3%) and soybean hulls (DM 81.7%, CP 10.2%, crude fat 0.8%, NDF 61.7%, and ADF 43.8%) in ratio of 50:50 during post-calving grazing period. Cows were provided with ad libitum hay (DM 76.0%, CP 9.4%, NDF 62.9%, and ADF 35.7%) from d 39 to weaning as forage availability declined in the fall. Cows were synchronized using the 7-d Co-Synch + controlled internal drug-release (Pfizer Animal Health, New York, NY) procedure [22] and artificially inseminated (AI; 78 ± 6 d post-calving). Ten days after AI, cows were exposed to 2 clean-up bulls for a 79 d breeding season. Pregnancy diagnosis to AI and overall pregnancy were performed 35 d and 98 d after AI. Pregnancy diagnosis was conducted by a trained technician with ultrasonography (Aloka 500 instrument, Wallingford, CT).

**Table 1:** Supplement composition and nutrient intake from treatment supplements containing Ca salts of saturated/monounsaturated fatty acids (CON) or polyunsaturated fatty acids (PUFA)

Item (Dry matter basis)	CON^a^	PUFA^b^
Ingredients, kg/cow/d		
Soybean hull	0.77	0.77
EnerGII	0.155	0
Prequel	0	0.08
Strata	0	0.08
Macronutrient intake, kg/cow/d		
Dry matter	0.9	0.9
Crude protein	0.07	0.07
Total fat	0.16	0.15
Fatty acid intake, g/d		
C16:0	57.86	14.43
C18:0	5.74	6.97
C18:1c9	44.69	24.41
C18:2n-6	13.60	33.24
C18:3n-3	1.32	2.24
C20:5n-3	0	6.41
C22:6n-3	0	4.12

^a^CON = cows were supplemented soybean hulls mixed with 155 g DM/cow/d EnerGII (Virtus Nutrition LLC, Corcoran, CA) for 77 ± 6 d prepartum

^b^PUFA = cows were supplemented soybean hulls mixed with 80 g DM/cow/d Strata + 80 g DM/cow/d Prequel (Virtus Nutrition LLC, Corcoran, CA) for 77 ± 6 d prepartum

**Table 2:** Nutritional and fatty acid profile of ingredients fed to cows during late gestation^a^

Item (Dry matter basis)	Forage	Soybean hull	EnerGII	Prequel	Strata
Dry matter, %	29.3	85.1	96.4	95.3	95.7
Crude protein, %	12.7	9.6	0	0	0
Neutral detergent fiber, %	56.6	71.7	–	–	–
Acid detergent fiber, %	35.2	51.8	–	–	–
Total fat, %	3.4	2.3	94.3	83.7	83.0
Total fatty acid, %	1.5	1.0	77.2	74.4	67.3
Fatty acid profile, % of total fatty acid					
C16:0	20.3	17.2	47.2	0.40	23.8
C18:0	2.32	7.68	4.28	3.88	7.52
C18:1c9	4.62	14.1	36.4	27.8	12.5
C18:2n-6	20.1	38.6	8.78	49.1	1.72
C18:3n-3	40.1	12.2	0.29	1.27	0.94
C20:5n-3	0	0	0	0	11.9
C22:6n-3	0	0	0	0	7.66

^a^EnerGII, Prequel, and Strata were from Virtus Nutrition LLC, Corcoran, CA

Cows were vaccinated at the initiation of the supplementation (d − 77) and the end of gestation (d − 18). On d − 77, cows were administered with 2 mL Leptoferm-5 (Zoetis, Florham Park, NJ), 1 mL Anaplasmosis vaccine (University Products L.L.C., Baton Rouge, LA), and 2 mL Auto. *M. bovis*. (Pinkeye vaccine customized by Newport Laboratories, Worthington, MN). In addition, 2 Patriot Insecticide Cattle Ear Tags (Bayer, Shawnee Mission, KS) and Ivermectin (Norbrook, UK) were applied to the cows. On d − 18, 5 mL Bovishield Gold FP5 VL5 HB (Zoetis), 5 mL Covexin 8 (Merck Animal Health), 7 mL MU-SE (Zoetis), and 2 mL ScourGuard 4KC (Zoetis) and Cylence (Bayer) were administered.

There were 12 cows from PUFA groups that were removed from the trial from late-supplementation to rebreed. One cow was removed at late-supplementation (d − 18) because of abortion. One cow was removed because of extremely poor BCS at calving. One was removed because of having twins. Five cows were detected open at calving. Three cows were removed because of loss of calves prior to when weigh-suckle-weigh was conducted. One cow from PUFA group died on December 2018. There was one cow from CON group was removed because of a stillborn birth. There were 5 and 8 heifer calves born from CON and PUFA cows, respectfully. Data from dams of heifer calves was included for analysis of cow performance data, while heifer calves were not included for analysis of calf birth BW, weaning BW, or mRNA expression.

Within 24 h after calving, calf birth BW was recorded and bull calves were castrated. Calves were provided with 1 mL BO-SE (Merck Animal Health), 20 mL Bovi-Sera (Colorado Serum Company, Denver, CO), and 1 mL Vitamin A/D (VetONE, Biose, ID) at birth. Calves were vaccinated on d 60 and d 172 with following: 2 mL Auto. *M. bovis*. (Newport Laboratories), 5 mL Covexin 8 (Merck Animal Health), 5 mL Bovishield Gold FP5 VL5 HB (Zoetis), 2 mL Pulmo-Guard PHM-1 (AgriLabs, St. Joseph, MO), and 1/100 dose of Synanthic (Boehringer Ingelheim Vetmedica, Duluth, GA). Two mL Myco-B One Dose (American Animal Health, Fort Worth, TX) and 2 mL Inforce 3 (Zoetis) were also administered on d 60. Calves were weighed and weaned at 186 ± 6 days of age. Pre-weaning growth was evaluated based on weaning BW and pre-weaning ADG.

### Sampling and analytical procedures

Blood samples were collected from cows at pre- (d − 77) and mid-supplementation (d − 42), and from cow/steer pairs within 1 week after calving (5 ± 2.4 d post-calving). Blood samples (10 mL) were collected from the jugular vein of cows and calves by using polypropylene tubes (BD Vacutainer) containing sodium heparin for plasma, and placed on ice. After centrifugation for 20 min at 2,000 × *g* and 4 °C, plasma was stored at − 80 °C until later analysis. For steer calves and their dams, individual plasma samples were thawed in 0–4 °C water bath [23]. A glass rod was used to stir the water in the tub for accelerating the thawing process. Thawed plasma samples that were from the same grazing group were individually added into a pooled centrifuge tube for each sampling time point. Within a pooled sample, each individual plasma sample accounted for the same percentage of the total 1.5 mL pooled unit.

Relative concentrations of fatty acids in pooled plasma samples were analyzed by Metabolomics Center at the Roy J. Carver Biotechnology Center (Urbana, IL, USA). Extraction was conducted twice on 100 μL of sample with 300 μL of methanol:chloroform (1:2) solution. Organic phase was collected, evaporated and hydrolyzed with 500 μL of 3 N methanolic HCL contained 2 g/L of butylated hydroxytoluene for 1 h at 85 °C. Samples then were cooled to room temperature and extracted twice with 500 μL of hexane. Organic phase was collected, evaporated under nitrogen and re-suspended in 100 μL. Samples were analyzed using a gas chromatography-mass spectrometry system (Agilent Inc., Palo Alto, CA, USA) consisting of an Agilent 7890B gas chromatograph and an Agilent 5977A MSD. Separation was performed on a HP-5MS (60 m × 0.25 mm I.D. and 320 μm film thickness) capillary column (Agilent J&W, Palo Alto, CA, USA). The inlet temperature was 220 °C, MSD interface temperature was 230 °C and the ion source temperature was adjusted to 230 °C. An aliquot of 1 μL injected in a splitless mode (20 mL/min for 0.75 min). The helium carrier gas was kept at a constant flow rate of 2 mL/min. The temperature program was: 2 min at 150 °C, followed by temperature increase of 5 °C per min to 300 °C for 3 min. The mass spectrometer operated in positive electron impact mode at 69.9 eV ionization energy at m/z 50–600 scan range. Target peaks were evaluated using AMDIS v2.71 and Mass Hunter Qualitative Analysis B.08.00 (Agilent Inc., Palo Alto, CA, USA) software.

Milk production was determined before breeding (64 ± 9 d postpartum) on a subset of 59 cow/ steer calf pairs (7–9 pairs per grazing group) via weigh-suckle-weigh (WSW) as described by Beal et al. [24]. Day postpartum, cow BW at calving and cow age were stratified across subset groups. Milk samples were hand stripped on a subset of cows (4 cows/pasture group; *n* = 32) during WSW for milk composition and fatty acid profile analysis. Milk samples for composition analysis were shipped in a cooler with ice packs underneath the samples. Milk composition was analyzed by Dairy Lab Service Inc. (Dubuque, IA). For the milk samples that were collected for fatty acid profile analysis, the top fat layer was collected after centrifugation at 20,000×*g* for 30 min at 4 °C [3] and stored at − 80 °C until shipping out to Cumberland Valley Analytical Service Inc. (CVAS; Waynesboro, PA, USA). The analysis was conducted as previously described [25, 26] with minor modifications. Briefly, approximately 1.6 mg of sample was weighed and extracted using a mixture of isopropanol and hexane (2:3, v/v). Extracts were collected and evaporated under nitrogen at 45 °C for about 8 min to dryness. Hexane and methyl acetate were used to dissolve lipid extracts, then fatty acid methyl esters (FAME) was synthesized by using methanolic sodium methoxide. The mixture was neutralized by oxalic acid, centrifuged and dried with calcium chloride. The FAME were separated and quantitated using PerkinElmer Clarus 590 (PerkinElmer, Shelton, CT) equipped with a fused-silica capillary column (SP-2560, 100 m × 0.25 mm i.d. with 0.2-μm film thickness; Supelco Inc., Bellefonte, PA) and a flame ionizationdetector (FID). Hydrogen was used as the carrier gas.

Feed samples including pasture and supplement samples were collected every 2 weeks during supplementation period for proximate and fatty acid profile analysis. Forage samples were hand-clipped at the beginning of each rotation. Samples of supplement during the comingled grazing period were collected every 2 weeks for proximate analysis. All feed samples were stored at − 20 °C until further processing. The pasture forage samples were composited on a monthly basis, while supplement samples were composited for the supplementation period. The samples that were for fatty acid profile analysis were freeze dried and ground through 1 mm screen using a Wiley mill (Arthur, H. Thomas, Philadelphia, PA). The rest of the samples that were for proximate analysis were composited and oven dried under 55 °C for at least 3 days, then ground through 1 mm screen using a Wiley mill. Ground samples were analyzed for DM (105 °C oven), crude protein (Leco TruMac, LECO Corporation, St. Joseph, MI), crude fat by using Ankom XT10 Fat Extractor (Ankom Technology, Macedon, NY), NDF and ADF by using an Ankom 200 Fiber Analyzer (Ankom Technology, Macedon, NY).

Fatty acid profile analysis of the feed samples was conducted by CVAS using method of Sukhija and Palmquist [27], which was modified as follows. One mL of internal standard (C13:0) was added to approximately 0.5 g of sample, followed by adding 3.0 mL of 5% methanolic HCL to sample. After vortex, sample was incubated in 70 °C water bath for 2 h. Sample was removed from water bath and cooled for 15 min. After cooling, 5.0 mL of 6% Potassium Carbonate and 1 mL of hexane were added to the sample. Sample was centrifuged at 752×*g* at room temperature for 10 min. The organic layer was transferred to gas chromatography autosampler vial. Fatty acid profile was analyzed on PerkinElmer Clarus 580, split/splitless capillary injector, and FID detector with helium as the carrier gas. The fused silica capillary column was Restek Rtx-2330, 30 m × 0.32 mm i.d. × 0.20 μm.

Muscle and adipose tissue biopsy samples for mRNA expression were collected from every steer calf at birth (5 ± 2 days of age) and from a subset of steers (*n* = 24, 3 from each grazing group) at 3 week. prior to weaning (165 ± 4 days of age). The 24 steers were selected based on their BW at 64 ± 9 days of age being representative to the group average. For muscle biopsies, an area over the *longissimus dorsi* muscle from the first lumbar vertebra region on the left side of the animal was clipped closely and scrubbed 3 times thoroughly with betadine surgical scrub, and rinsed with 70% alcohol. Lidocaine was administered subcutaneously and intramuscularly (5–10 mL) over the biopsy site. After 10 min of lidocaine administration, a biopsy core of muscle (100–200 mg) was removed by using a biopsy needle (Bard MAGNUM; 12 gauge × 16 cm). The initial *Longissimus* muscle biopsy was taken 5 cm cranial to the hook bone half way between the axis and transverse processes of the lumbar vertebrae. After completing the biopsy, pressure was applied with sterile gauze to stop any external bleeding, the surrounding area was cleansed with sterile saline to remove blood, the incision was closed with a synthetic absorbable tissue adhesive Vetbond (3 M Animal Care Products, St. Paul, MN, USA), and a topical antibiotic ointment was applied. Each subsequent biopsy was taken 5 cm cranial to the previous biopsy site. For subcutaneous adipose tissue biopsies, an area of approximately 15 cm × 15 cm over one side of the tail-head area was cleaned the same as muscle biopsy procedure. Lidocaine was administered similarly as muscle biopsy. Thereafter, a 3 to 4-cm incision was made with a sterile surgical blade and the skin was pulled back using forceps, exposing the subcutaneous adipose tissue. Samples of adipose (1 to 3 g) were taken using forceps and a single-use sterile scalpel blade. The closure of the incision was conducted similarly as in muscle biopsy. Each subsequent biopsy was collected from the opposite side of the tail-head region. Biopsy samples were immediately frozen in liquid nitrogen and stored at − 80 °C for later mRNA expression analysis.

### mRNA expression

Biopsy tissue was weighed (50 mg for muscle tissue, 200 mg for adipose tissue) and RNA extracted using Qiazol Lysis Reagent (Qiagen Inc., Valencia, CA, USA) following the manufacturer’s instruction. Genomic DNA was removed by using RNeasy Mini Kit (Qiagen Inc., Valencia, CA, USA). Concentration of RNA was measured with NanoDrop ND-1000 spectrophotometer (NanoDrop Technologies, Wilmington, DE). Quality of RNA was assessed using a 2100 Bioanalyzer (Agilent Technologies Inc., Santa Clara, CA) to calculate a RNA integrity value (RIN) for each sample. Values for RIN range from 1 to 10 (low to high quality) based on the area of 18S and 28S rRNA area and the height of the 28S rRNA peak. Extracted RNA had mean RIN values of 8.8 ± 0.48 for muscle tissue and 7.4 ± 1.61 for adipose. A portion of RNA was diluted to 100 mg/L using DNase/RNase free water prior to reverse transcription.

Complementary DNA (cDNA) was synthesized using 600 ng RNA, 54 μL DNase/RNase free water and 6 μL Random Primers (Roche). The mixture was incubated at 65 °C for 5 min and placed on ice for 3 min. Fifty-four μL of master mix contained 24 μL 5X First-Strand Buffer, 6 μL Oligo dT18, 12 μL 10 mmol/L dNTP mix (Thermo Fisher Scientific), 1.5 μL RevertAid Reverse Transcriptase (200 U/μL, EP0442, Thermo Fisher Scientific), and 0.75 μL RiboLock RNase Inhibitor (40 U/μL, EO0381, Thermo Fisher Scientific). The reaction was carried out in a SureCycler 8800 Thermal Cycler (Agilent Technologies Inc., Santa Clara, CA, USA) using the following temperature program: 25 °C for 5 min, 42 °C for 60 min and 70 °C for 5 min. Resulting cDNA was then diluted 1:4 with DNase/RNase free water.

The primers used for Quantitative PCR (qPCR) are listed in Supplemental Table 1. The design and evaluation of the primers that had no references were conducted according to the method reported by Bionaz and Loor [28]. Sequence results for the DNA products of the designed primers are presented in Supplemental Table 2. The sequencing product was confirmed by BLASTN at the National Center for Biotechnology Information database (NCBI).

Quantitative PCR was performed using 4 μL diluted cDNA combined with 6 μL of a mixture composed of 5 μL of Sybr Green Fast Mix ROX (QuantaBio Inc., Gaithersburg, MD, USA), 0.4 μL of forward primer, 0.4 μL of reverse primer, and 0.2 μL of DNase/RNase free water. All cDNA samples were analyzed in triplicate and 7-point relative standard curve plus the negative control (DNase/RNase free water instead of cDNA template) were used. The amplification protocol was as follow: 2 min at 50 °C, 5 min at 95 °C, 40 cycles at 95 °C for 5 s, 60 °C for 30 s. After amplification, a melting curve analysis was performed over a range of 60–95 °C to verify that a single PCR product was generated at the end of essay. The data were calculated with the QuantStudio Real-Time PCR Software (version 1.3, Thermo Fisher Scientific). The final data were normalized using the geometric mean of internal control genes: *GAPDH*, *ACTB*, and *RPLP0* for muscle samples, *ACTB*, *BRPS2*, and *SLC35BC* for adipose tissue samples. Treatment effect was analyzed for the abundance of the internal control genes; there were no treatment effects on abundance of any of the five internal control genes. Information about qPCR performance of the genes are presented in Supplemental Tables 3 and 4.

### Statistical analysis

Cow grazing group was considered the experimental unit for all response variables. Data, except AI pregnancy rates, were analyzed using the MIXED procedure of SAS (version 9.4; SAS Institute Inc., Cary, NC, USA). Outliers were checked by using Proc Reg procedure of SAS removing data with a studentized t > 3.0 prior to analysis. A random statement of cow group nested within treatment was included in all of the analysis under MIXED procedure when individual animal observational data was used. Treatment means were separated by the least square means function of SAS. The model for cow BW and BCS included treatment as fixed effect and cow age as covariate. The model for milk yield included the fixed effect of treatment, random effect cow group nested within treatment, and included cow age, cow milk expected progeny differences (EPD) and days postpartum as covariates. The model for calf plasma fatty acid relative concentrations, cow milk composition and milk fatty acid profile included treatment as a fixed effect and day-postpartum as a covariate. The model for steer weaning BW and pre-weaning ADG included the fixed effects of treatment and the covariate of weaning BW EPD. Data concerning relative mRNA abundance but not normally distributed were transformed with a logarithmic function (LOG) or a Box-Cox family of power transformation to improve normality. For the analysis of relative mRNA abundance, treatment, time (either at-birth or at-weaning), and the interaction between treatment and time were used as fixed effects, group nested within treatment and sire were included as random effects. Repeated measure analysis of SAS was used for the analysis of cow plasma fatty acid relative concentrations. The model includes treatment, time (mid-supplementation or at-calving), and the interaction between treatment and time as fixed effects, and the concentration of the corresponding fatty acid at pre-supplementation as a covariate. The repeated measure was also used for the analysis of forage availability, and the model included treatment, time, and the interaction between treatment and time as fixed effects. Heterogeneous compound symmetry was used as the covariance structure for cow plasma fatty acid relative concentration and forage availability based on Akaike information criterion. The GLIMMIX procedure of SAS was used for the analysis of AI pregnancy rates, with treatment as fixed effect and age as a covariate in the model. A random statement including cow group nested within treatment, sire, and AI technician were included for the analysis of AI pregnancy rates. Overall pregnancy rate was initially analyzed with the same model as AI pregnancy rate; however, the data did not converge as some of the groups had 100% pregnancy rate. Thus, the MIXED procedure of SAS was used for the analysis of overall pregnancy rate, with each group being the observational unit. Significance was declared at *P* ≤ 0.05, and tendencies were declared from 0.05 < *P* ≤ 0.10.

## Results

In the current study, cows rotationally grazed on tall fescue pastures with no difference in forage availability (CON: 2015 kg DM/ha, PUFA: 1957 kg DM/ha at the end of rotation; *P* = 0.28) during the supplementation period.

### Relative concentrations of selected plasma fatty acids

There were treatment × time interactions (*P* ≤ 0.05; Table 3) for cow plasma concentrations of α-linolenic acid (C18:3n-3; ALA) and C20:4n-6, where the concentrations of ALA and C20:4n-6 decreased to a greater extent from mid-gestation to calving for PUFA compared with CON. The concentration of C20:3n-6 tended (*P* = 0.07) to decrease to a greater extent from mid-gestation to calving for CON cows compared with PUFA cows. Dams supplemented with PUFA had greater (*P* ≤ 0.01) concentrations of C20:0, EPA, and DHA, but tended (*P* = 0.09) to have lesser concentration of C18:1c9 compared with CON dams. Steers from CON dams had greater (*P* ≤ 0.01; Table 4) concentrations of C16:1c9, C18:1c9, ALA, and C20:3n-6, while lesser (*P* = 0.01) concentration of EPA compared with steers from PUFA dams. Steers from PUFA dams tended (*P* = 0.09) to have greater concentration of DHA at birth.

**Table 3:** Effects of late gestation supplementation of Ca salts of saturated/monounsaturated fatty acids (CON) or polyunsaturated fatty acids (PUFA) on relative concentrations of selected plasma fatty acids in cows^1^

Item	Mid-sup^2^		At-calving^3^		SEM	*P*-values^6^		
	CON^4^	PUFA^5^	CON	PUFA		Trt	Time	Trt × time
C15:0	39.6	42.1	27.4	31.8	1.95	0.15	< 0.01	0.58
C16:0	1257	1183	1380	1362	58.1	0.44	0.04	0.65
C16:1c9	10.2	16.1	28.4	35.7	4.95	0.12	< 0.01	0.86
C17:0	62.2	61.6	49.0	59.0	4.60	0.27	0.11	0.26
C18:0	1387	1396	1009	1048	49.8	0.70	< 0.01	0.63
C18:1c9	961	800	1225	1077	92.4	0.09	0.03	0.95
C18:2n-6	1190	1176	751	875	50.7	0.39	< 0.01	0.11
C18:3n-3	262^a^	252^a^	173^b^	98^c^	20.9	0.08	< 0.01	0.05
C20:0	2.7	4.4	3.1	4.5	0.38	< 0.01	0.61	0.78
C20:3n-6	141	89	96	73	13.0	0.05	< 0.01	0.07
C20:4n-6	195^a^	213^a^	49^b^	31^c^	8.5	0.97	< 0.01	0.03
C20:5n-3^7^	40.2	93.2	33	57.5	–	< 0.01	0.03	0.50
C22:6n-3	4.8	26.4	7.2	20.7	4.09	0.01	0.59	0.22

^1^Data are presented as relative concentration per 100 μL plasma to internal standard C23:0; means within a row with different superscript differ at *P* ≤ 0.05

^2^Mid-supplementation was d − 42 of the experiment

^3^At-calving plasma samples were collected from dams 5 ± 2.4 d post-calving

^4^CON = cows were supplemented soybean hulls mixed with 155 g DM/cow/d EnerGII (Virtus Nutrition LLC, Corcoran, CA) for 77 ± 6 d prepartum

^5^PUFA = cows were supplemented soybean hulls mixed with 80 g DM/cow/d Strata + 80 g DM/cow/d Prequel (Virtus Nutrition LLC, Corcoran, CA) for 77 ± 6 d prepartum

^6^*Trt* Treatment effect, *Trt × time* Interaction between treatment and time

^7^Data were transformed for statistical analysis because of non-normal distribution; Means are presented after back-transformation

**Table 4:** Effects of late gestation supplementation of Ca salts of saturated/monounsaturated fatty acids (CON) or polyunsaturated fatty acids (PUFA) on relative concentrations of selected plasma fatty acids in steers at birth^a^

Item	CON^b^	PUFA^c^	SEM	*P-*value
C15:0	21.7	19.2	2.14	0.29
C16:0	1101	1030	104.1	0.53
C16:1c9	23.4	15.0	1.77	0.01
C17:0	28.6	27.5	2.47	0.69
C18:0	1036	1021	64.0	0.83
C18:1c9	1132	881	40.4	< 0.01
C18:2n-6	772	855	67.4	0.27
C18:3n-3	176	98	12.2	< 0.01
C20:0	3.6	4.3	0.48	0.20
C20:3n-6	48.4	31.7	3.06	< 0.01
C20:4n-6	146	138	13.0	0.58
C20:5n-3	36.3	56.7	5.22	0.01
C22:6n-3	9.0	18.8	4.78	0.09

^a^Data are presented as relative concentration per 100 μL plasma to internal standard C23:0

^b^CON = cows were supplemented soybean hulls mixed with 155 g DM/cow/d EnerGII (Virtus Nutrition LLC, Corcoran, CA) for 77 ± 6 d prepartum

^c^PUFA = cows were supplemented soybean hulls mixed with 80 g DM/cow/d Strata + 80 g DM/cow/d Prequel (Virtus Nutrition LLC, Corcoran, CA) for 77 ± 6 d prepartum

### Cow performance

Cow BW and BCS were not affected (*P* ≥ 0.34; Table 5) by late gestation supplementation at any of the time points from the start of the supplementation through weaning. The length of supplementation was not different (CON 80.3 , PUFA 81.7 d; *P* ≥ 0.51). Gestation length of the cows was not affected by late gestation supplementation (*P* = 0.46; Table 8). The pregnancy rates for AI and overall breeding season of the cows were not different (*P* ≥ 0.35; Table 6).

**Table 5:** Effects of fatty acid supplementation on cow body weight and body condition score^a^

Item	CON^b^	PUFA^c^	SEM	*P*-value
Cow BW, kg				
Initial (d − 77)	604	597	12.4	0.62
Mid supplementation (d − 42)	642	632	12.6	0.43
Pre calving (d − 18)	652	642	12.7	0.46
Calving (5 ± 2.4 d post-calving)	597	589	13.1	0.57
AI (d 78)	610	595	14.5	0.34
AI pregnancy determination (d 133)	597	587	15.6	0.52
Weaning (d 186)	540	526	14.5	0.39
Cow BCS				
Initial	6.2	6.2	0.14	0.89
Mid supplementation	6.3	6.4	0.13	0.47
Pre calving	6.4	6.3	0.16	0.36
Calving	6.1	6.2	0.13	0.45
AI	5.5	5.6	0.14	0.41
AI pregnancy determination	5.4	5.3	0.15	0.77
Weaning	4.8	4.8	0.18	0.99

^a^*BW* Body weight; *AI* Artificial insemination; *BCS* Body condition score

^b^CON = cows were supplemented soybean hulls mixed with 155 g DM/cow/d EnerGII (Virtus Nutrition LLC, Corcoran, CA) for 77 ± 6 d prepartum

^c^PUFA = cows were supplemented soybean hulls mixed with 80 g DM/cow/d Strata + 80 g DM/cow/d Prequel (Virtus Nutrition LLC, Corcoran, CA) for 77 ± 6 d prepartum

**Table 6:** Effects of late gestation supplementation of Ca salts of saturated/monounsaturated fatty acids (CON) or polyunsaturated fatty acids (PUFA) on milk production^a^ and cow reproductive performance

Item	CON^b^	PUFA^c^	SEM	*P*-value
Milk production, kg/d	8.6	9.4	0.75	0.33
Composition				
Fat, %	2.4	2.3	0.36	0.75
Protein, %	3.1	3.0	0.10	0.38
Lactose, %	4.8	4.8	0.05	0.23
Other solids, %	6.1	6.0	0.06	0.35
Total solids, %	11.7	11.4	0.36	0.48
MUN, mg/dL	14.0	14.5	0.89	0.59
AI pregnancy, %	75	64	–	0.35
Overall pregnancy, %	93	97	4.3	0.46

^a^Milk production was determined via weigh-suckle-weigh technique at 64 ± 9 d postpartum on a subset of 7–9 pairs/group; Milk composition conducted on a subset of cows (4 cows/group) at 64 ± 9 d postpartum; *MUN* Milk urea nitrogen; *AI* Artificial insemination

^b^CON = cows were supplemented soybean hulls mixed with 155 g DM/cow/d EnerGII (Virtus Nutrition LLC, Corcoran, CA) for 77 ± 6 d prepartum

^c^PUFA = cows were supplemented soybean hulls mixed with 80 g DM/cow/d Strata + 80 g DM/cow/d Prequel (Virtus Nutrition LLC, Corcoran, CA) for 77 ± 6 d prepartum

### Milk production, composition, and fatty acid profile

Milk production of the cows was not different (*P* = 0.33; Table 6) between treatments. The milk fat, protein, lactose, total solids, or milk urea nitrogen (MUN) was not affected (*P* ≥ 0.23) by supplements during late gestation. Fatty acid profile of milk fat is shown in Table 7. The concentration of DHA was greater (*P* = 0.02) in milk fat of the dams supplemented with PUFA. Concentrations of other fatty acids in milk fat were not different (*P* ≥ 0.15).

**Table 7:** Effects of late gestation supplementation of Ca salts of saturated/monounsaturated fatty acids (CON) or polyunsaturated fatty acids (PUFA) on fatty acid profile of milk fat

Item	CON^a^	PUFA^b^	SEM	*P*-value
Fatty acid, % of total fatty acids				
C4:0	3.76	3.73	0.133	0.80
C6:0	1.64	1.60	0.064	0.52
C8:0	0.97	0.93	0.057	0.53
C10:0	2.06	1.97	0.170	0.64
C12:0	2.37	2.32	0.216	0.85
C14:0	8.05	7.74	0.446	0.51
C15:0	1.01	1.07	0.043	0.24
C16:0^c^	22.7	21.6	–	0.19
C16:1c9	1.41	1.40	0.082	0.88
C17:0	0.65	0.66	0.025	0.52
C18:0	11.38	11.31	0.799	0.93
C18:1 t4	0.04	0.04	0.003	0.62
C18:1 t5	0.03	0.03	0.003	0.76
C18:1 t6, 8	0.47	0.49	0.024	0.44
C18:1 t9	0.36	0.37	0.015	0.47
C18:1 t11	4.62	4.70	0.220	0.72
C18:1 t12	0.39	0.41	0.020	0.58
C18:1c9	23.83	24.65	1.099	0.49
C18:1c11	0.42	0.40	0.027	0.58
C18:1c12	0.12	0.12	0.007	0.84
C18:1 t16	0.32	0.32	0.012	0.59
C18:2 t11, c15	0.11	0.09	0.018	0.52
C18:2n-6	2.58	2.72	0.140	0.37
C20:0	0.17	0.17	0.014	0.76
C20:1c11	0.05	0.05	0.003	0.66
C18:3n-3	0.50	0.52	0.030	0.36
C18:2c9, t11	2.22	2.28	0.140	0.66
C20:2n-6	0.02	0.02	0.002	0.48
C22:0	0.06	0.06	0.006	0.69
C20:3n-6	0.08	0.08	0.006	0.16
C20:4n-6	0.13	0.12	0.006	0.15
C20:5n-3	0.04	0.04	0.004	0.52
C22:5n-3	0.08	0.08	0.005	0.86
C22:6n-3	0.01	0.02	0.004	0.02
Total n-3	0.61	0.66	0.033	0.18
Total n-6	2.82	2.93	0.141	0.46

^a^CON = cows were supplemented soybean hulls mixed with 155 g DM/cow/d EnerGII (Virtus Nutrition LLC, Corcoran, CA) for 77 ± 6 d prepartum

^b^PUFA = cows were supplemented soybean hulls mixed with 80 g DM/cow/d Strata + 80 g DM/cow/d Prequel (Virtus Nutrition LLC, Corcoran, CA) for 77 ± 6 d prepartum

^c^Data were transformed for statistical analysis because of non-normal distribution; Means are presented after back-transformation

### Pre-weaning growth performance

Growth performance of the steer progeny during pre-weaning stage is presented in Table 8. Birth BW of the steers was not affected (*P* = 0.75) by different fatty acid profile of maternal supplements. However, weaning BW of the steers from CON dams was 11 kg greater (*P* = 0.05), which led to a tendency (*P* = 0.06) for CON steers to have greater pre-weaning ADG compared with the steers from PUFA dams.

**Table 8:** Effects of late gestation supplementation of Ca salts of saturated/monounsaturated fatty acids (CON) or polyunsaturated fatty acids (PUFA) on calf performance

Item^a^	CON^b^	PUFA^c^	SEM	*P*-value
Gestation length, d	275.3	276.2	1.26	0.46
Birth BW, kg	37.5	37.3	0.62	0.75
Weaning BW, kg	206	195	4.4	0.05
Pre-weaning ADG, kg/d	0.91	0.85	0.023	0.06
Weaning age, d	186	185	1.5	0.57

^a^*BW* Body weight, *ADG* Average daily gain

^b^CON = cows were supplemented soybean hulls mixed with 155 g DM/cow/d EnerGII (Virtus Nutrition LLC, Corcoran, CA) for 77 ± 6 d prepartum

^c^PUFA = cows were supplemented soybean hulls mixed with 80 g DM/cow/d Strata + 80 g DM/cow/d Prequel (Virtus Nutrition LLC, Corcoran, CA) for 77 ± 6 d prepartum

### Relative mRNA expression in Longissimus muscle

Relative mRNA expression of the genes that regulate early development of muscle and adipose tissue in LM is presented in Table 9. There were treatment × time interactions (*P* ≤ 0.04) for the mRNA expression of *MYH7* and *C/EBPβ*. The mRNA expression of *MYH7* and *C/EBPβ* increased to a greater extent from birth to weaning for PUFA steers compared with CON. The expression of *MYF5* tended (*P* = 0.07) to decrease more from birth to weaning for CON steers compared with PUFA. The expression of *MYOG* tended (*P* = 0.08) to be greater in CON steers than in PUFA steers at both birth and weaning. From birth to weaning, the expression of *MYH1*, *MYH2*, *MYF6*, *MYOD1*, *C/EBPα*, *ZFP423*, *FABP4*, *PPARG*, and *PPARGC1A* were increased (*P* ≤ 0.02) in LM. However, the expressions of *PAX7* and *MEF2C* decreased from birth to weaning (*P* < 0.01).

**Table 9:** Relative expression of genes regulating myogenesis and adipogenesis in *Longissimus* muscle of the steers born from dams supplemented with Ca salts of saturated/monounsaturated fatty acids (CON) or polyunsaturated fatty acids (PUFA) during late gestation^1^

Gene^5^	Birth		Weaning		*P*-value^4^		
	CON^2^	PUFA^3^	CON	PUFA	Trt	Time	Trt × time
*MYH1*	0.854	0.897	1.324	1.212	0.85	< 0.01	0.50
*MYH2*	1.014	0.883	1.367	1.159	0.18	0.01	0.90
*MYH7*	1.032^b^	0.923^c^	1.136^a^	1.173^a^	0.18	< 0.01	0.02
*PAX7*	1.134	1.067	0.971	0.979	0.34	< 0.01	0.18
*MYF5*	1.418	0.987	0.915	0.894	0.08	0.01	0.07
*MYF6*	0.887	0.627	1.694	1.544	0.18	< 0.01	0.23
*MYOD1*	0.979	0.884	1.386	1.174	0.34	0.02	0.80
*MYOG*	1.053	0.830	1.280	1.042	0.08	0.05	0.88
*MEF2C*	1.504	1.323	0.976	0.969	0.56	< 0.01	0.50
*C/EBPα*	0.505	0.531	1.905	2.102	0.59	< 0.01	0.86
*C/EBPβ*	0.186^c^	0.163^c^	0.422^b^	0.566^a^	0.46	< 0.01	0.04
*ZFP423*	0.836	0.735	1.764	1.844	0.69	< 0.01	0.26
*FABP4*	0.154	0.271	0.738	0.481	0.88	0.02	0.25
*PPARG*	0.316	0.298	0.727	0.679	0.63	< 0.01	0.98
*AGPAT1*	1.245	1.147	1.225	1.376	0.81	0.23	0.16
*PPARGC1A*	0.609	0.472	0.911	0.995	0.77	0.01	0.37

^1^Means are back-transformed if transformation was conducted; means within a row with different superscript differ at *P* ≤ 0.05

^2^CON = cows were supplemented soybean hulls mixed with 155 g DM/cow/d EnerGII (Virtus Nutrition LLC, Corcoran, CA) for 77 ± 6 d prepartum

^3^PUFA = cows were supplemented soybean hulls mixed with 80 g DM/cow/d Strata + 80 g DM/cow/d Prequel (Virtus Nutrition LLC, Corcoran, CA) for 77 ± 6 d prepartum

^4^*Trt* Treatment effect, *Trt × time* Interaction between treatment and time

^5^*MYH1* Myosin heavy chain 1, *MYH2* Myosin heavy chain 2, *MYH7* Myosin heavy chain 7, *PAX7* Paired box protein 7, *MYF5* Myogenic factor 5, *MYF6* Myogenic factor 6, *MYOD1* Myogenic differentiation 1, *MYOG* Myogenin, *MEF2C* Myocyte enhancer factor 2C, *C/EBPα* CCAAT enhancer binding protein alpha, *C/EBPβ* CCAAT enhancer binding protein beta, *ZFP423* Zinc finger protein 423, *FABP4* Fatty acid binding protein 4, *PPARG* Peroxisome proliferator activated receptor gamma, *AGPAT1* Acylglycerol phosphate acyltransferase 1, *PPARGC1A* PPARG coactivator 1 alpha

### Relative mRNA expression in subcutaneous adipose tissue

Relative mRNA expression of the genes that regulate the development of adipose tissue in subcutaneous tissue is presented in Table 10. Maternal supplementation of CON increased (*P* = 0.02) the mRNA expression of *ZFP423* in the steer progeny at both birth and weaning compared with maternal supplementation of PUFA. Relative mRNA expression of *C/EBPβ* tended (*P* = 0.08) to decrease to a greater extent from birth to weaning for CON compared with PUFA steers. However, mRNA expression of *SCD* tended (*P* = 0.08) to decrease to a greater extent from birth to weaning for PUFA compared with CON steers. From birth to weaning, the relative mRNA expression of *C/EBPα*, *PPARGC1A*, *FASN*, *SREBP1*, and *ACACA* were decreased (*P* ≤ 0.02) in subcutaneous adipose tissue.

**Table 10:** Relative expression of genes regulating adipogenesis in subcutaneous adipose tissue of the steers born from dams supplemented with Ca salts of saturated/monounsaturated fatty acids (CON) or polyunsaturated fatty acids (PUFA) during late gestation^1^

Gene^5^	Birth		Weaning		*P*-value^4^		
	CON^2^	PUFA^3^	CON	PUFA	Trt	Time	Trt × time
*C/EBPα*	1.094	0.903	0.830	0.727	0.22	0.02	0.62
*C/EBPβ*	0.319	0.266	0.241	0.267	0.68	0.09	0.08
*ZFP423*	0.978	0.812	1.396	1.149	0.02	< 0.01	0.56
*FABP4*	0.922	0.992	1.095	1.071	0.88	0.17	0.61
*PPARG*	1.044	0.956	0.793	0.868	0.96	0.07	0.37
*PPARGC1A*	0.572	0.435	0.102	0.136	0.99	< 0.01	0.44
*SCD*	0.771	1.096	0.238	0.131	0.70	< 0.01	0.08
*FASN*	0.897	0.964	0.202	0.131	0.57	< 0.01	0.33
*SREBP1*	1.065	0.986	0.764	0.786	0.84	< 0.01	0.54
*ACACA*	0.946	1.151	0.182	0.144	0.94	< 0.01	0.37
*ADFP*	0.943	0.735	0.720	0.808	0.69	0.57	0.25

^1^Means are back-transformed if transformation was conducted

^2^CON = cows were supplemented soybean hulls mixed with 155 g DM/cow/d EnerGII (Virtus Nutrition LLC, Corcoran, CA) for 77 ± 6 d prepartum

^3^PUFA = cows were supplemented soybean hulls mixed with 80 g DM/cow/d Strata + 80 g DM/cow/d Prequel (Virtus Nutrition LLC, Corcoran, CA) for 77 ± 6 d prepartum

^4^*Trt* Treatment effect, *Trt × time* Interaction between treatment and time

^5^*SCD* Stearoyl-CoA desaturase, *FASN* Fatty acid synthase, *SREBP1* Sterol regulatory element binding transcription factor 1, *ACACA* Acetyl-CoA carboxylase alpha, *ADFP* Adipose differentiation-related protein

## Discussion

Forage availability between treatments during supplementation period did not differ, and cow/calf pairs were comingled and managed as a common group following supplementation period. Therefore, any differences in animal responses would be attributed to different fatty acid profile in the maternal supplements during late gestation.

### Cow performance

Relative concentrations of plasma fatty acids were modified by supplements correspondingly with the fatty acid profile in the supplements, which indicates that fatty acid supplementation successfully modified the circulating fatty acids in cow plasma. In addition, supplementing Ca salts of fatty acids 3 times/week. has been documented to result in stable circulating fatty acid concentrations [29]. Essential fatty acids circulating in maternal plasma become available to the developing fetus after being transferred across the placenta [10]. In the current study, EFAs like ALA and C20:3n-6 were increased in CON dams at calving, while EPA and DHA were increased in PUFA dams throughout the supplementation period. Similarly, other studies that have supplemented dams with different fat sources reported modified circulation of fatty acids corresponding with the feeding source [3, 30, 31]. In addition, EFAs supplemented to dairy cows during transition period were found elevated in skeletal muscle and fat tissues of neonate calves [32], which also demonstrates the potential effects of maternal EFAs on progeny muscle and adipose development. It is recognized that maternal circulating EFAs like linoleic acid, ALA, arachidonic acid, and DHA can modulate fetal growth and development [33]. However, BW and BCS of the cows were not affected by different fatty acid profile in supplements. This is consistent with previous studies that supplemented cows in an isocaloric and isonitrogenous manner [2, 34].

Milk production was not affected by different fatty acid profiles in late gestation supplements. Cows from both treatments produced approximately 9 kg/d of milk at d 64 ± 9 after calving. For milk composition, the only difference was milk from PUFA dams had greater concentration of DHA in milk fat. All fatty acids that have 18 or longer chain are derived from preformed fatty acids from circulating plasma lipids [35], which means increased concentration of DHA in milk of PUFA cows should originate from absorption or reserves. Given that milk samples were collected approximately 60 d after supplementation, the increased concentration of DHA could be due to mobilization of reserves during negative energy balance, which was supported by decreased BCS from calving to AI pregnancy determination. However, this difference was minor because of the low concentration of DHA in milk from both treatments. Consistent with our results in milk evaluations, late gestation supplementation of fatty acids did not change either milk yield or composition in ewes [11]. However, it was also reported that late gestation of EPA + DHA did not change EPA or DHA concentrations in ewes’ milk at 30 d after parturition [3]. The possible reasons for no differences in EPA or DHA concentrations in ewes could be low supplementation levels and tissue storage of fatty acids were depleted after 30 d of supplementation. In addition, milk production was not affected by fat supplementation during the last 62 d of gestation of primiparous beef cows [36]. No differences in milk production and composition were expected for the current study, as supplementation was terminated at calving and the WSW and milk sampling were conducted 64 d after supplementation.

Reproductive performance of the cows was not statistically different between cows fed different fatty acid supplements in late gestation. However, authors acknowledge that the current study is not powered for reproductive performance. Research that investigated prepartum fat supplementation have reported varied responses on reproductive performances. Heifers fed late gestation fat supplementation had increased subsequent pregnancy rates [34, 37], especially under situations with limited forage availability. Supplementation was terminated at calving in the current study, and forage availability was sufficient before breeding season, which together could be the reason for no differences. Improvement in reproductive performance were commonly reported when the fat supplementation takes place during postpartum [38–40], in which fat supplementation, especially EFAs, could have impacts on the balance of reproductive hormones [41, 42]. Studies with greater number of animals are needed for detecting differences in reproductive performance of cows supplemented with different fatty acid profile during late gestation.

### Calf performance

It has been well recognized that environmental factors including maternal nutrition affect the growth and development of the subsequent progeny [13, 43, 44]. Recent promising effects including greater finishing phase growth performance, greater hot carcass weight and marbling score in beef cattle [2] and greater finished BW in sheep [4] indicate positive fetal programming potential of maternal fatty acid supplementation during late gestation. The development of skeletal muscle and adipose tissue is important for beef cattle. However, the mechanisms of maternal supplementation differing in fatty acid profile affecting the transcription of critical genes in muscle and adipose tissue have not been investigated in beef cattle.

Birth BW of the steers was not affected by maternal supplementation differing in fatty acid profile, despite relative concentrations of calf plasma fatty acids at birth being modified by treatments. Steers from CON dams had greater concentrations of C16:1c9, C18:1c9, ALA, and C20:3n-6, while PUFA steers had greater concentrations of EPA and DHA. In sheep, oleic acid tended to be greater in lambs from dams that had palmitic acid enriched supplementation during late gestation, while no differences were detected for ALA, EPA, or DHA [3]. However, in the current study, greater plasma concentrations of C18:1c9 and ALA in CON calves, C20:3n-6, EPA, and DHA in PUFA calves were consistent with corresponding concentrations in their dams. No difference in birth BW of progeny is consistent with previous studies that supplemented ruminant dams with isocaloric and isonitrogenous rumen-protected saturated/monounsaturated fatty acids or PUFA [2, 11], sunflower seeds high in LA [45, 46], and safflower seeds, raw soybeans, or sunflower seeds [34] during late gestation.

Despite no differences in birth BW and dam milk yield, CON steers had greater weaning BW and tended to have greater pre-weaning ADG compared with PUFA steers. Steer response to the greater milk DHA in PUFA should be limited as the concentration of DHA was extremely low in milk. Inconsistent with the current study, studies have reported that pre-weaning performance of the calves or lambs from dams supplemented with either palm fatty acid distillate or PUFA was not different [2, 11]. Previous studies [2, 11] have used both sexes but neither reported treatment × sex interactions. Thus, authors do not believe the difference in results was due to this study using only male calves. Having different outcomes could also be due to different calving systems and supplementation rates. In Marques et al. [2], cows were under a spring-calving system and were fed 10.1 kg/cow grass-alfalfa hay as their basal diet; and in Coleman et al. [11], ewes were limit-fed a mixed ration in pens. While in the current study, fall-calving cows were grazing on abundant tall-fescue pastures. Compared with Marques et al. [2], cows in the current study were exposed to greater temperatures (July to September for the late gestation), which could potentially cause heat stress to the cows and lead to negative programming effects on the subsequent lactation of the dams, postnatal growth performance and immune function of the offspring [47]. Additionally, it is reported that ergot alkaloids found in endophyte tall-fescue can cause vasoconstriction in grazing animals [48], which could potentially impact the fatty acid circulation and transferring to the fetus in the current study. Compared with 190 g/d of Prequel/Strata in Marques et al. [2], 160 g/d of Prequel/Strata were fed to cows in the current study. The ratio of Prequel to Strata in the two studies was the same, while the concentration of EPA + DHA (33.3%) in Strata of Marques et al. [2] was greater than the current study (18.5%) and the product label (16% minimum). This resulted in 26 g/d of EPA/DHA delivered to cows in Marques et al. [2] and 10.5 g/d in the current study.

During late gestation, restriction of maternal nutrition reduces muscle fiber size, but has little impact on muscle fiber number [49, 50]. However, nutritional deficiency during late gestation reduces the density of satellite cells, which could also negatively affect postnatal muscle growth [51]. Therefore, it is important to provide reasonable nutrients for optimal fetal muscle development and subsequent postnatal growth performance. According to the mRNA expression analysis on muscle and subcutaneous adipose tissue collected at birth and weaning, myogenesis and adipogenesis of the steers could be modified by maternal supplements.

In *longissimus* muscle, there tended to be a treatment × time interaction for the mRNA expression of *MYF5*, in which CON steers had the greatest expression of *MYF5* at birth. The increase in expression of *MYF5* activates all other myogenic regulatory factors (*MYOD*, *MYOG* and *MYF6*) [52]. It was documented that *MYF5* is directly regulated by *PAX7*, and transcription of *MYF5* varies dependent on the levels of *PAX7* [53, 54]. In the current study, no treatment or treatment × time effect was detected for the expression of *PAX7*. Other factors like signals of the Wingless and Int family and Sonic hedgehog also directly induce *MYF5* expression [55], but are more important during early embryonic stage [52]. The tendency of greater expression of *MYF5* at birth in CON steers indicates they potentially had greater myogenesis in LM in the neonatal stage. Greater myogenesis in CON steers was also supported by the tendency of having greater expression of *MYOG* in LM. Gene *MYOD* is an early myogenic marker, which is critical in regulating the maturation of secondary muscle fibers [56]. The current study also indicates *MYOG* was downregulated by maternal supplementation of PUFA compared with CON. Inconsistent with our finding for *MYOG*, Brandão et al. [5] reported that the expression of *MYOG* in LM at birth was greater in calves from dams supplemented with Ca salts of soybean oil than those from dams supplemented with saturated fat. However, Brandão et al. [5] did not find any differences on offspring growth performance during pre-weaning period. Studies on C2C12 myoblasts [57, 58] indicate that overdosage of EPA and DHA could inhibit myogenesis and down-regulate myogenic genes, including *MYOG*, *MYF6*, *MYOD*, and *PAX7*. The supplementation level of EPA and DHA (10.5 g/d) in current study should not have resulted in overdosage. However, results from this study indicate the development of secondary muscle fibers in CON steers could be greater during pre-weaning period compared with PUFA steers. Mechanism for PUFA steers having lower mRNA expression of *MYF5* and *MYOD* than CON needs further investigation. In muscle, *MYH1*, *MYH2*, and *MYH7* are associated with myosin heavy chains IIx, IIa, and I, respectively [59]. Myosin heavy chain I is a slow isoform also called beta-myosin heavy chain. The expression of *MYH7* was detected in developing muscles of embryo and fetus, and in type 1 muscle fibers and ventricles in adults [60]. There was a treatment × time interaction detected for the expression of *MYH7*, which indicates the formation of myosin heavy chain I in PUFA steers was slow at birth and increased at time of weaning.

Intramuscular fat is key for palatability of beef because it contributes to flavor and juiciness [13]. The sequential adipogenesis in different depots provides an opportunity to specifically enhance the intramuscular adipocyte formation during late gestation, neonatal stage, and early postnatal stage (earlier than 250 days of age) [50]. Nutritional manipulations, like maternal supplementation of nutrients or bioactive compounds, during these time windows are expected to be able to modify intramuscular adipogenesis [13, 61]. The mRNA expression of *CAAT/enhancer binding protein β* (*C/EBPβ*) in LM was affected by the treatment × time interaction, in which the expression increased to a greater extent from birth to weaning for PUFA compared with CON steers. The essential genes that regulate adipogenesis include *C/EBPα*, *C/EBPβ*, *PPARG*, *ZFP423*, and *FABP4* [57]. The transcription factors *C/EBPβ* and *δ* are induced by adipogenic stimuli at the initiation of adipogenesis, which then lead to the expression of *PPARG* [62]. For adipogenesis, *PPARG* is essential and indispensable [63]. Therefore, *C/EBPβ* is critical for early stage of adipocyte differentiation. Our data indicates that the intramuscular adipocyte differentiation could be increased at weaning by maternal supplementation of PUFA during late gestation, which might lead to greater formation of marbling. However, further research following steers all the way to slaughter is still needed.

Subcutaneous fat is not desirable, because it negatively impacts feed efficiency and devalues the carcass [64]. Research investigating fat supplementation on lipid metabolism of ewes [3], lambs [65], dairy cows [66], and young bulls [67] has been conducted, but the effect of maternal fat supplementation on lipid metabolism in offspring tissues is scarcely studied. Zinc-finger protein 423 was reported to induce *PPARG* expression and the adipogenic commitment of progenitor cells [68]. Increased expression of *ZFP423* in CON steers at birth and weaning indicates greater adipogenic commitment in subcutaneous adipose tissue, which might lead to greater 12th rib fat thickness and negatively affect feed efficiency and carcass yield grade. The 12th rib fat of the calves was not affected by maternal supplementation of calcium salts of PUFA during late gestation under a spring-calving system [7]. The current study did not measure 12th rib fat thickness at weaning, and further research is needed to investigate maternal fat supplementation of different fatty acid profile on 12th rib fat thickness of the carcass at slaughter. Adipogenesis is triggered when *PPARG* is activated by *C/EBPβ* and *C/EBPδ* [64]. Stearoyl-CoA desaturase (*SCD*) is required for the biosynthesis of monounsaturated fatty acids [69]. Mature *SREBP1* activates transcription of *FASN* and *SCD* [69]. Treatment × time interaction detected for *C/EBPβ* and *SCD* in the current study might indicate modified adipogenesis by maternal supplementations differing in fatty acid profile. Coleman et al. [65] reported finishing lambs from dams fed EPA and DHA during late gestation had decreased hepatic mRNA expression of *SCD* compared with palm fatty acid distillate, while no effect on subcutaneous adipose mRNA expression of *SCD* was detected. Further investigation is needed for the regulation of maternal fatty acid supplementation on mRNA expression of adipogenic genes and lipid metabolism in subcutaneous adipose tissue.

## Conclusions

Late gestation supplementation with differing fatty acid profiles did not affect cow BW, BCS, or rebreeding pregnancy rates. For steer progeny, maternal supplementation enriched in saturated and monounsaturated fatty acids increased weaning BW compared with PUFA, which could be attributed to upregulated mRNA expression of myogenic genes during pre-weaning period, especially at birth. These findings encourage further investigation of maternal supplementation of different fatty acid profile on subsequent progeny growth and production performance and mRNA expression of myogenic and adipogenic genes under different production systems.

## Supplementary Information

**Additional file 1: Supplemental Table 1.** GenBank accession number, sequence and amplicon size of primers used for mRNA expression by qPCR. **Supplemental Table 2.** Sequencing results of PCR products of designed primer. **Supplemental Table 3.** qPCR performance of the genes analyzed in Longissimus muscle. **Supplemental Table 4.** qPCR performance of the genes analyzed in adipose tissue.

## Data Availability

The datasets during and/or analyzed during the current study are available from the corresponding authors on reasonable request.

## Abbreviations

*ACACA*: Acetyl-CoA carboxylase alpha
ADF: Acid detergent fiber
*ADFP*: Adipose differentiation-related protein
ALA: Alpha-linolenic acid
FAME: Fatty acid methyl esters
ADG: Average daily gain
AI: Artificial insemination
BCS: Body condition score
BW: Body weight
*C/EBPα*: CCAAT enhancer binding protein alpha
*C/EBPβ*: CCAAT enhancer binding protein beta
CON: Supplement rich in saturated and monounsaturated fatty acids
CP: Crude protein
DHA: Docosahexaenoic acid
DM: Dry matter
EFAs: Essential fatty acids
EPA: Eicosapentaenoic acid
EPD: Expected progeny differences
*FABP4*: Fatty acid binding protein 4
*FASN*: Fatty acid synthase
*AGPAT1*: Acylglycerol phosphate acyltransferase 1
HCW: hot carcass weight
LM: Longissimus muscle
*MEF2C*: Myocyte enhancer factor 2C
MUN: Milk urea nitrogen
*MYF5*: Myogenic factor 5
*MYF6*: Myogenic factor 6
*MYH1*: Myosin heavy chain 1
*MYH2*: Myosin heavy chain 2
*MYH7*: Myosin heavy chain 7
*MYOD1*: Myogenic differentiation 1
*MYOG*: Myogenin
NCBI: National Center for Biotechnology Information database
NDF: Neutral detergent fiber
*PAX7*: Paired box protein 7
*PPARG*: Peroxisome proliferator activated receptor gamma
*PPARGC1A*: PPARG coactivator 1 alpha
PUFA: Supplement rich in polyunsaturated fatty acids
qPCR: Quantitative PCR
*SCD*: Stearoyl-CoA desaturase
*SREBP1*: Sterol regulatory element binding transcription factor 1
WSW: Weigh-suckle-weigh
*ZFP423*: Zinc finger protein 423
