# Supplementary material for: Effects of supplements differing in fatty acid profile to late gestational beef cows on cow performance, calf growth performance, and mRNA expression of genes associated with myogenesis and adipogenesis
Source: J Anim Sci Biotechnol. 2021 Jun 14;12:67. doi: 10.1186/s40104-021-00588-w (PMC8201839; doi:10.1186/s40104-021-00588-w)
Supplement: Supplementary file 1 — Additional file 1: Supplemental Table 1. GenBank accession number, sequence and amplicon size of primers used for mRNA expression by qPCR. Supplemental Table 2. Sequencing results of PCR products of designed primer. Supplemental Table 3. qPCR performance of the genes analyzed in Longissimus muscle. Supplemental Table 4. qPCR performance of the genes analyzed in adipose tissue. [file 40104_2021_588_MOESM1_ESM.docx]

Supplemental Table 1. GenBank accession number, sequence and amplicon size of primers used for mRNA expression by qPCR

| Accession number | Gene^1^ | Direction | Primer sequences (5’ to 3’) | Amplicon length (bp) |
| --- | --- | --- | --- | --- |
| NM_001034034.2 | *GAPDH* [1] | F78 | AAGGTCGGAGTGAACGGATTC | 98 |
|  |  | R175 | AAGGGGTCATTGATGGCGAC |  |
| NM_173979.3 | *ACTB* [2] | F862 | GCCCTGAGGCTCTCTTCCA | 100 |
|  |  | R961 | CGGATGTCGACGTCACACTT |  |
| NM_001012682.1 | *RPLP0* [3] | F657 | CAACCCTGAAGTGCTTGACAT | 227 |
|  |  | R883 | AGGCAGATGGATCAGCCA |  |
| NM_001033613 | *BRPS2* [4] | F320 | GGAGCATCCCTGAAGGATGA | 101 |
|  |  | R420 | TCCCCGATAGCAACAAACG |  |
| BC103464 | *SLC35B2* [5] | F894 | ACATTGCTTTCGACAGCTTCAC | 95 |
|  |  | R988 | GAAGAGATTGACCCCAAACATCA |  |
| NM_174116.1 | *MYF5* [6] | F892 | CCTCTAGTTCCAGGCTCATCTA | 90 |
|  |  | R981 | ACCTCCTTCCTCCTGTGTAATA |  |
| NM_181811.2 | *MYF6* | F591 | AAGAAAATCTTGAGGGTGCGGA | 96 |
|  |  | R686 | CCTTGGCAGTTATCACGAGC |  |
| NM_001111325 | *MYOG* [6] | F222 | GGCGTGTAAGGTGTGTAAG | 85 |
|  |  | R306 | CTTCTTGAGTCTGCGCTTCT |  |
| NM_001040478.2 | *MYOD1* | F827 | AACTGTTCCGACGGCATGAT | 105 |
|  |  | R931 | CCGGGGTTCGTTGGGC |  |
| XM_015460690.2 | *PAX7* | F435 | AGATCGAGGAGTACAAGAGGGA | 112 |
|  |  | R545 | ATCGAACTCACTGAGGGCACG |  |
| NM_174117.1 | *MYH1* | F3924 | AGGTGAATTCTCACGCCAGC | 136 |
|  |  | R4059 | CAGGGCACTCTTGGCCTTTA |  |
| NM_001166227.1 | *MYH2* | F3979 | GCTTGCAGACTGAATCTGGTG | 149 |
|  |  | R4127 | GGCATTCTTGGCCTTTATCTCC |  |
| NM_174727.1 | *MYH7* | F2160 | TTCCGGCAGAGGTATCGAAT | 128 |
|  |  | R2287 | TGGCCGAACTTATACTGGTTGTG |  |
| NM_001046113 | *MEF2C* [6] | F703 | CCTGATGCAGACGATTCAGTAG | 123 |
|  |  | R825 | AAAGTTGGGAGGTGGAACAG |  |
| NM_001101893.1 | *ZFP423* [7] | F240 | GGATTCCTCCGTGACAGCA | 120 |
|  |  | R359 | TCGTCCTCATTCCTCTCCTCT |  |
| NM_181024.2 | *PPARG* [8] | F135 | CCAAATATCGGTGGGAGTCG | 101 |
|  |  | R235 | ACAGCGAAGGGCTCACTCTC |  |
| NM_176784.2 | *C/EBPα* | F385 | TTCAACGACGAGTTCCTGGC | 107 |
|  |  | R491 | CCCGGGTAGTCAAAGTCGTT |  |
| NM_176788.1 | *C/EBPβ* [7] | F333 | CGGGCAGCACCACGACTTCC | 106 |
|  |  | R438 | CCCCAGTCGGCCCAGACTCA |  |
| NM_174314.2 | *FABP4* [9] | F401 | TGGTGCTGGAATGTGTCATGA | 101 |
|  |  | R501 | TGGAGTTCGATGCAAACGTC |  |
| NM_177945 | *PPARGC1A* [10] | F2001 | GTACCAGCACGAAAGGCTCAA | 120 |
|  |  | R2120 | ATCACACGGCGCTCTTCAA |  |
| CR552737 | *FASN* [11] | F6383 | ACCTCGTGAAGGCTGTGACTCA | 92 |
|  |  | R6474 | TGAGTCGAGGCCAAGGTCTGGAA |  |
| NM_001113302.1 | *SREBP1* [12] | F2858 | TACCTGCAGCTTCTCCATCA | 145 |
|  |  | R3002 | CACCAATGGGTACAGCCTCT |  |
| BT029909.1 | *ADFP* [13] | F911 | GTCTGTCCTGGCTGGAGTGGAAGAG | 150 |
|  |  | R1060 | TGTTGGACAGGAGGGTGTGGCA |  |
| NM_173959.4 | *SCD* [14] | F809 | TCCTGTTGTTGTGCTTCATCC | 101 |
|  |  | R909 | GGCATAACGGAATAAGGTGGC |  |
| NM_177518 | *AGPAT1* [15] | F563 | TGCCATCAGTGTCATGTCTG | 86 |
|  |  | R648 | GGTTTCTCGTGCCCTCAG |  |
| NM_174224.2 | *ACACA* | F182 | TGTGAAGTATCCTTCTGGAGGT | 99 |
|  |  | R280 | CTTCCAAAAAGAACTCAGAGACC |  |

^1^*GAPDH* glyceraldehyde-3-phosphate dehydrogenase, *ACTB* actin beta, *RPLP0* ribosomal protein lateral stalk subunit P0, *BRPS2* ribosomal protein S2, *SLC35B2* solute carrier family 35 member B2, *MYF5* Myogenic factor 5, *MYF6* Myogenic factor 6, *MYOG* Myogenin, *MYOD1* Myogenic differentiation 1, *PAX7* Paired box protein 7, *MYH1* Myosin heavy chain 1, *MYH2* Myosin heavy chain 2, *MYH7* Myosin heavy chain 7, *MEF2C* Myocyte enhancer factor 2C, *ZFP423* Zinc finger protein 423, *PPARG* Peroxisome proliferator activated receptor gamma, *C/EBPα* CCAAT enhancer binding protein alpha, *C/EBPβ* CCAAT enhancer binding protein beta, *FABP4* Fatty acid binding protein 4, *PPARGC1A* PPARG coactivator 1 alpha, *FASN* Fatty acid synthase, *SREBP1* Sterol regulatory element binding transcription factor 1, *ADFP* Adipose differentiation-related protein, *SCD* Stearoyl-CoA desaturase, *AGPAT1* Acyl-glycerol phosphate acyltransferase 1, *ACACA* Acetyl-CoA carboxylase alpha

Supplemental Table 2. Sequencing results of PCR products of designed primer

| Gene | Sequence |
| --- | --- |
| *MYF6* | AAGAAAATCTTGAGGGTGCGGATTTCCTGCGCACCTGCAGCTCCCAGTGGCCAAGTGTTTCGGATCATTCCAGGGGGCTCGTGATAACTGCCAAGG |
| *MYOD1* | AACTGTTCCGACGGCATGATGGACTACAGCGGCCCCCCGAGTGGTGCCCGGCGGCGGAACTGCTACGACCGCACTTACTACAGCGAGGCGCCCAACGAACCCCGG |
| *PAX7* | AGATCGAGGAGTACAAGAGGGAAAACCCGGGCATGTTTAGCTGGGAGATCCGAGACCGACTGCTGAAGGACGGGCACTGCGACCGCAGCACCGTGCCCTCAGTGAGTTCGAT |
| *MYH1* | AGGTGAATTCTCACGCCAGCTAGATGAAAAGGATGCACTCGTTTCTCAGCTCTCAAGGGGCAAACAAGCATTCACACAACAGATTGAAGAACTGAAAAGGCAGCTGGAAGAGGAGATAAAGGCCAAGAGTGCCCTG |
| *MYH2* | GCTTGCAGACTGAATCTGGTGAATTTTCACGTCAGCTAGATGAGAAAGAAGCTCTGGTGTCTCAGTTGTCAAGGGGCAAACAAGCATTTACTCAACAGATTGAGGAATTAAAGAGGCAACTTGAAGAGGAGATAAAGGCCAAGAATGCC |
| *MYH7* | TTCCGGCAGAGGTATCGAATCCTGAACCCAGCAGCCATCCCCGAGGGCCAGTTCATTGACAGCAGGAAAGGGGCAGAGAAGTTGCTGGGCTCCCTGGACATTGACCACAACCAGTATAAGTTCGGCCA |
| *C/EBPα* | TTCAACGACGAGTTCCTGGCCGACCTGTTCCAACACAGCCGGCAGCAGGAGAAGGCCAAGGCGGCCGCGGCCCCCGCAGGAGGCGGCAACGACTTTGACTACCCGGG |
| *ACACA* | TGTGAAGTATCCTTCTGGAGGTTTTTGCCTCCCATACGCGACCTGAAGACGGACAAGCAGACGTTGGAAGCAGAGAGGTCTCTGAGTTCTTTTTGGAAG |

Supplemental Table 3. qPCR performance of the genes analyzed in *Longissimus* muscle

| Gene | Median Ct^1^ | Median ∆Ct^2^ | Slope^3^ | (R^2^)^4^ | Efficiency^5^ | Relative mRNA abundance^6^ |
| --- | --- | --- | --- | --- | --- | --- |
| *MYF6* | 22.022 | 4.241 | -3.367 | 0.989 | 1.982 | 0.00797 |
| *MYOD1* | 23.624 | 5.913 | -3.322 | 0.990 | 2.000 | 0.00240 |
| *MYOG* | 23.069 | 5.367 | -3.583 | 0.989 | 1.901 | 0.00460 |
| *PAX7* | 29.332 | 11.560 | -3.398 | 0.995 | 1.969 | 0.00006 |
| *MYF5* | 26.603 | 8.880 | -3.167 | 0.988 | 2.069 | 0.00023 |
| *GPAT1* | 26.539 | 8.755 | -3.512 | 0.991 | 1.926 | 0.00047 |
| *PPARGC1A* | 24.204 | 6.482 | -3.533 | 0.995 | 1.919 | 0.00212 |
| *MYH1* | 15.793 | -2.009 | -3.395 | 0.983 | 1.970 | 0.56580 |
| *MYH2* | 16.967 | -0.782 | -3.544 | 0.987 | 1.915 | 0.24073 |
| *MYH7* | 17.528 | -0.245 | -3.319 | 0.994 | 2.001 | 0.17163 |
| *PPARG* | 28.847 | 11.047 | -3.231 | 0.974 | 2.039 | 0.00006 |
| *ZFP423* | 24.948 | 7.157 | -3.541 | 0.985 | 1.916 | 0.00138 |
| *MEF2C* | 24.440 | 6.555 | -3.251 | 0.991 | 2.030 | 0.00140 |
| *C/EBPα* | 28.612 | 11.009 | -3.276 | 0.979 | 2.020 | 0.00006 |
| *C/EBPβ* | 28.350 | 10.775 | -3.317 | 0.977 | 2.002 | 0.00008 |
| *FABP4* | 25.539 | 7.704 | -3.58 | 0.990 | 1.903 | 0.00102 |

The data were calculated based on the qPCR conducted for at-birth and at-weaning

^1^ The median is calculated considering all time points and all steers

^2^ The median of ∆Ct is calculated as [Ct gene – geometrical mean of Ct internal controls] for each time point and each steer

^3^ Slope of the standard curve

^4^ R2 stands for the coefficient of determination of the standard curve

^5^ Efficiency is calculated as [10(-1 / Slope)]

^6^ Relative mRNA abundance = 1/ Efficiency Median ∆Ct

Supplemental Table 4. qPCR performance of the genes analyzed in adipose tissue

| Gene | Median Ct^1^ | Median ∆Ct^2^ | Slope^3^ | (R^2^)^4^ | Efficiency^5^ | Relative mRNA abundance^6^ |
| --- | --- | --- | --- | --- | --- | --- |
| *FASN* | 20.570 | 0.685 | -3.458 | 0.996 | 1.946 | 0.63361 |
| *SREBP1* | 22.209 | 2.095 | -3.531 | 0.993 | 1.920 | 0.25506 |
| *PPARG* | 22.552 | 2.606 | -3.492 | 0.996 | 1.934 | 0.17933 |
| *FABP4* | 15.401 | -4.437 | -3.523 | 0.994 | 1.922 | 18.17106 |
| *ADFP* | 22.384 | 2.530 | -3.503 | 0.994 | 1.930 | 0.18952 |
| *PPARGC1A* | 28.436 | 7.647 | -3.568 | 0.982 | 1.907 | 0.00719 |
| *ZFP423* | 23.320 | 3.432 | -3.514 | 0.990 | 1.926 | 0.10551 |
| *SCD* | 19.165 | -0.962 | -3.527 | 0.994 | 1.921 | 1.87412 |
| *C/EBPα* | 22.346 | 2.461 | -3.495 | 0.995 | 1.933 | 0.19767 |
| *C/EBPβ* | 27.889 | 7.695 | -3.504 | 0.991 | 1.929 | 0.00637 |
| *ACACA* | 23.568 | 3.464 | -3.530 | 0.997 | 1.920 | 0.10439 |

The data were calculated based on the qPCR conducted for at-birth and at-weaning

^1^ The median is calculated considering all time points and all steers

^2^ The median of ∆Ct is calculated as [Ct gene – geometrical mean of Ct internal controls] for each time point and each steer

^3^ Slope of the standard curve

^4^ R2 stands for the coefficient of determination of the standard curve

^5^ Efficiency is calculated as [10(-1 / Slope)]

^6^ Relative mRNA abundance = 1/ Efficiency Median ∆Ct

References:

1. Kadegowda AKG, Bionaz M, Thering B, Piperova LS, Erdman RA, Loor JJ. Identification of internal control genes for quantitative polymerase chain reaction in mammary tissue of lactating cows receiving lipid supplements. J Dairy Sci. 2009;92:2007–19. https://doi.org/10.3168/jds.2008-1655

2. Goselink RMA, van Baal J, Widjaja HCA, Dekker RA, Zom RLG, de Veth MJ, et al. Effect of rumen-protected choline supplementation on liver and adipose gene expression during the transition period in dairy cattle. J Dairy Sci. 2013;96:1102–16. https://doi.org/10.3168/jds.2012-5396

3. Wang YH, Byrne KA, Reverter A, Harper GS, Taniguchi M, McWilliam SM, et al. Transcriptional profiling of skeletal muscle tissue from two breeds of cattle. Mamm Genome. 2005;16:201–10. https://doi.org/10.1007/s00335-004-2419-8

4. Rocco SM, McNamara JP. Regulation of bovine adipose tissue metabolism during lactation. 7. Metabolism and gene expression as a function of genetic merit and dietary energy intake. J Dairy Sci. 2013;96:3108–19. https://doi.org/10.3168/jds.2012-6097

5. Mukesh M, Bionaz M, Graugnard DE, Drackley JK, Loor JJ. Adipose tissue depots of Holstein cows are immune responsive: Inflammatory gene expression in vitro. Domest Anim Endocrinol. 2010;38:168–78. https://doi.org/10.1016/j.domaniend.2009.10.001

6. Su X, Wang Y, Li A, Zan L, Wang H. Neudesin neurotrophic factor promotes bovine preadipocyte differentiation and inhibits myoblast myogenesis. Animals. 2019;9:1109. https://doi.org/10.3390/ani9121109

7. Duarte MS, Paulino PVR, Das AK, Wei S, Serão NVL, Fu X, et al. Enhancement of adipogenesis and fibrogenesis in skeletal muscle of Wagyu compared with Angus cattle. J Anim Sci. 2013;91:2938–46. https://doi.org/10.2527/jas.2012-5892

8. Ji P, Osorio JS, Drackley JK, Loor JJ. Overfeeding a moderate energy diet prepartum does not impair bovine subcutaneous adipose tissue insulin signal transduction and induces marked changes in peripartal gene network expression. J Dairy Sci. 2012;95:4333–51. https://doi.org/10.3168/jds.2011-5079

9. Segers JR, Loor JJ, Moisá SJ, Gonzalez D, Shike DW. Effects of protein and fat concentration in coproduct-based growing calf diets on adipogenic and lipogenic gene expression, blood metabolites, and carcass composition. J Anim Sci. 2017;95:2767–81. https://doi.org/10.2527/jas.2017.1446

10. Thering BJ, Bionaz M, Loor JJ. Long-chain fatty acid effects on peroxisome proliferator-activated receptor-α-regulated genes in Madin-Darby bovine kidney cells: Optimization of culture conditions using palmitate. J Dairy Sci. 2009;92:2027–37. https://doi.org/10.3168/jds.2008-1749

11. Bionaz M, Loor JJ. Gene networks driving bovine milk fat synthesis during the lactation cycle. BMC Genomics. 2008;9:366.

12. Taniguchi M, Guan LL, Zhang B, Dodson M V., Okine E, Moore SS. Adipogenesis of bovine perimuscular preadipocytes. Biochem Biophys Res Commun. 2008;366:54–9. https://doi.org/10.1186/1471-2164-9-366

13. Hiller B, Hocquette JF, Cassar-Malek I, Nuernberg G, Nuernberg K. Dietary n-3 PUFA affect lipid metabolism and tissue function-related genes in bovine muscle. Br J Nutr. 2012;108:858–63. https://doi.org/10.1017/S0007114511006179

14. Khan MJ, Jacometo CB, Graugnard DE, Corrêa MN, Schmitt E, Cardoso F, et al. Overfeeding dairy cattle during late-pregnancy alters hepatic pparα-regulated pathways including hepatokines: Impact on metabolism and peripheral insulin sensitivity. Gene Regul Syst Bio. 2014;2014:97–111. https://doi.org/10.4137/GRSB.S14116

15. Jeong J, Kwon EG, Im SK, Seo KS, Baik M. Expression of fat deposition and fat removal genes is associated with intramuscular fat content in longissimus dorsi muscle of Korean cattle steers. J Anim Sci. 2012;90:2044–53. https://doi.org/10.2527/jas.2011-4753
